# 50 Years of Research on the Psychology of Sport Injury: A Consensus Statement

**DOI:** 10.1007/s40279-024-02045-w

**Published:** 2024-06-11

**Authors:** Ulrika Tranaeus, Adam Gledhill, Urban Johnson, Leslie Podlog, Ross Wadey, Diane Wiese Bjornstal, Andreas Ivarsson

**Affiliations:** 1 Stockholm Sports Trauma Research Centre, FIFA Medical Centre of Excellence, The Swedish School of Sport and Health Sciences, GIH, Box 5626, 114 86 Stockholm, Sweden; 2 Department of Physiology, Nutrition, Biomechanics, Sport Performance and Exercise Research and Innovation Centre-Stockholm, SPERIC-S, The Swedish School of Sport and Health Sciences, Stockholm, Sweden; 3 Unit of Intervention and Implementation for Worker Health, Institute of Environmental Medicine, Karolinska Institutet, Stockholm, Sweden; 4 Carnegie School of Sport, Leeds Beckett University, Leeds, UK; 5 School of Health and Welfare, Halmstad University, Halmstad, Sweden; 6 School of Kinesiology and Physical Activity Sciences, Faculté de Médecine, Université de Montréal, Montreal, QC Canada; 7 Centre de Recherche, CHU-Saint Justine, Montreal, QC Canada; 8 St Mary’s University, Twickenham, London, UK; 9 School of Kinesiology, University of Minnesota Twin Cities, Minneapolis, USA; 10 Department of Sport Science and Physical Education, University of Agder, Kristiansand, Norway

## Abstract

Factors influencing sport injury risk, rehabilitation outcomes, and return to sport processes have been the focus in various research disciplines (sports medicine, psychology and sociology). One discipline, with over 50 years of scholarship, is the psychology of sport injury. Despite the research in this field, there is no evidence-based consensus to inform professional practice. The aim of this original and timely consensus statement is to summarise psychological sport injury research and provide consensus recommendations for sport practitioners seeking to implement psychological principles into clinical practice. A total of seven experts with extensive experience outlined the consensus objectives and identified three psychology of sport injury sub-domains: risk, rehabilitation and return to sport. The researchers, grouped in pairs, prepared initial drafts of assigned sub-domains. The group met in Stockholm, and the three texts were merged into a draft and revised in an iterative process. Stress responses are the strongest psychological risk factor for acute injuries. Intra- and interpersonal factors, as well as sociocultural factors, are demonstrated psychosocial risk factors for overuse injuries. Stress management and mindfulness interventions to prevent injuries have been successfully implemented. The rehabilitation process may influence athlete’s cognitive, emotional, and behavioural responses. Social support, mindfulness, acceptance-based practices, and cognitive-behavioural based intervention programs reduce negative reactions. Return to sport includes various stages and different trajectories. Returning athletes typically experience concerns regarding competence, autonomy, and relatedness. It is recommended that athletes focus on the physical, technical, and psychological demands of their sport as they progress to increasingly intense activities. Interdisciplinary collaboration (e.g., sports medicine and psychology) would be beneficial in enhancing clinical practice and improving athlete outcomes.

## Key Points

**Table:** 

Regarding injury risk, form strong relationships with your athletes and colleagues, screen athletes frequently for psychosocial stress and perceived recovery and consider stakeholder education around organisational injury risk factors.
Regarding rehabilitation, incorporate elements of mindfulness and acceptance-based practices and cognitive-behavioural based programs to improve coping and provide time and space to contribute as much social support as possible.
Regarding return to sport, utilise strategies that facilitate athletes’ sense of competence, autonomy, relatedness and facilitate clear lines of communication between athletes, coaches and medical staff.

## Introduction

The consequences of sport injury are significant and meaningful. On an individual level, injured athletes are at risk for a variety of mental health issues, including clinical anxiety, disordered eating, depression and suicidal ideation [1, 2]. Sport injury is also a leading cause of athletic career termination [3] which can have negative implications for athletes’ post-career physical [4] and psychological well-being [5, 6]. On an interpersonal level, injury can impair interactions between injured athletes and relevant others, such as coaches, teammates and family members [7, 8]. Finally, on a broader social level, injuries have potential legal, ethical or economic implications, in for instance, situations where athlete health and safety come into conflict/question or where teams and athletes may incur financial costs [9]. From a competitive standpoint, injuries are correlated with fewer competitions/games won [10]—and particularly those in professional sport—are highly costly (e.g. £45 million per year owing to reduced performance in the English Premier League) [11]. In sum, injury has salient implications for injured performers and injury stakeholders.

Given the negative consequences of injury, it is understandable that significant attention has been given to factors influencing injury risk, rehabilitation and return to sport. Guided by a medical model approach, much emphasis has been placed on physical, physiological and biomechanical factors associated with injury risk, prevention, rehabilitation and return to sport. Medically informed research has fundamentally formed the foundation of prominent guidelines within the sports medicine domain (e.g. risk for injury [12], rehabilitation [13], return to sport [14]). Acknowledgment of the psychological aspects of sport injury, has until recently, however, been given far less recognition. This lack of recognition is somewhat surprising, given that over 50 years of sport injury psychology research has consistently demonstrated the influential role of psychological factors in injury risk, rehabilitation and return to sport. Unfortunately, very few updated evidence-based consensus guidelines exist regarding the psychological aspects of injury [15]. Further, sport medicine practitioners often acknowledge that they are under-prepared for dealing with the psychological aspects of sport injury [16]. It is therefore timely to produce consensus guidelines regarding psychological factors implicated in injury risk, rehabilitation and return to sport.

The aim of this original and timely consensus statement is to summarise psychological sport injury research and provide consensus recommendations regarding integration of psychological principles into professional practice. To this end, we adopt a life-span perspective on severe musculoskeletal injury, that is, we examine key psychological issues pertaining to injury risk, rehabilitation, and return to sport and/or retirement from sport. Our focus is on musculoskeletal injury,^1^ given the prevalence of such injuries across multiple levels of sport [19–21] and the fact that much of the psychology of sport injury research has examined this form of injury [22, 23]. This consensus statement is relevant to sports medicine providers, coaches, administrators, injured athletes and students seeking to adopt a psychologically informed approach to mitigating injury risk and improving rehabilitation and return to sport outcomes.

## Methods

This consensus statement involved a series of digital meetings spanning May 2022 to July 2023 to co-construct the aim of this consensus statement, set the agenda for the formal meetings to follow, review and take stock of the sport injury psychology literature to enable an informed and enriched discussion, and establish working groups for each of the subsections (i.e., preinjury, rehabilitation and return to sport). Following the digital meetings, in-person meetings were then held in December 2022 at the Swedish School of Sport and Health Sciences, in the Stockholm Sports Trauma Research Centre, a FIFA Medical Centre of Excellence. Here, the aims of the formal meetings were to further discuss and debate literature, with each panel member acting as a theoretical sounding board for one another by posing challenging questions and encouraging reflection on the evidence and consensus across literature. We also sought to co-construct psychologically informed evidence-based guidelines [24].

### Panel Selection

Scholars with extensive research and applied practical experience in the psychology of sport injury were identified by the lead author (UT) and invited to a working group whose aim was to discuss relevant content and a methodological approach for producing the consensus statement. To be a member of the working group, potential experts were required to have: (i) a minimum of 10 years of applied experience working with injured athletes, and (ii) a minimum of 20 publications on sport injury psychology. Collectively, the seven researchers had more than 130 years of academic experience, and over 150 peer reviewed publications, book chapters and books on the psychological aspects of sport injury [25–27]. The group’s citations ranged from 1043 to 5774 [mean (m) = 3767] on Google Scholar. The author team included seven researchers (five males and two females) representing six universities in Canada, Sweden, the UK and the USA.

### Equity, Diversity and Inclusion Statement

The author team consisted of men and women from different countries, continents and academic degrees. On the basis of the aforementioned criteria, all consensus team members were considered experts in the psychological aspects of sport injury, thus minimising the inclusion of junior researchers and narrowing the disciplinary expertise.

### Evidence Reviews

We sought to include historical papers dating back to the 1980s, when research on the psychology of sport injury started to burgeon, up to recent work from the present day, reflecting state-of-the art knowledge. We decided to incorporate both formative research (i.e. published work from the 1980s and 1990s) as well as more recent publications (i.e. within the past 15 years) to capture salient findings across the 50 years of psychological injury scholarship. Our search strategy involved an online search of the following databases: PsycINFO, PsycARTICLES, SPORTDiscus, Web of Science Core Collection and the Cochrane Library. We used keywords (‘injury’, ‘sport’, ‘psychology’, ‘risk’, ‘prevention’, ‘rehabilitation’, ‘return’ and ‘retirement’) that captured sport injury experiences. In searching for articles across the databases, our intent was not to be exhaustive, but rather, to retrieve literature relevant to our objective. We also manually explored germane journals in the sport psychology, sport medicine and sport sociology literature.

### Consensus Process

The group outlined the aims and work process and identified three temporal themes for inclusion in the consensus statement: injury risk, rehabilitation, and return to sport/retirement at the outset of the process. Experts were grouped into pairs and were tasked by the lead author with identifying key content for one of the three temporal themes to which they had been assigned according to their predominant expertise. Prior to the Stockholm meeting, each pair circulated a draft text with a list of initial references pertaining to their temporal theme.

Subsequently, five of the seven experts in the work group met in Stockholm for 3.5 days. One person attended 1.5 days and one person participated through a digital link. During the Stockholm meeting, the workflow proceeded in the following manner: First, the entire working group discussed the overall objectives of the consensus statement and potential content for each of the three temporal themes. As the intent of our consensus statement was not to provide an exhaustive or systematic/narrative review of literature—but rather, to synthesize knowledge of three key themes on the psychology of sport injury and to offer practitioner recommendations—we did not apply specific guidelines or criteria in the selection of particular content or articles. Second, following discussion with the overall group, each pair updated and revised their initial draft text. Third, each pair presented their content to the entire group allowing for discussion, debate and agreement on issues or points raised. Such discussion iteratively took place until consensus was reached on content. Fourth, the various sections were merged into a full initial draft, which was subsequently edited, and revised to facilitate coherence between the three sub-sections. When all experts agreed on the text, the manuscript was sent to three sports medicine practitioners (i.e. one physiotherapist and two orthopaedic surgeons) with over 20 years’ experience working with injured athletes to facilitate knowledge transfer and exchange. The practitioners provided feedback on the manuscript’s content and practical relevance. To provide recommendations for evidence, a modified version of Grading of Recommendations, Assessment, Development and Evaluation (GRADE) [28] was used to rate the quality of evidence on the basis of study design, consistency of evidence and directness of evidence (Table 1). Finally, the comments from the invited reviewers were discussed and the manuscript was revised until all authors agreed on the final version. In sum, our consensus process was rigorous and as robust as those described by scholars undertaking previous consensus statements published in high-impact journals [14].

**Table 1:** Evidence in research on the psychology of sport injuries

Topic	Study design quality	Consistency of evidence	Directness of evidence
Psychological risk factors for acute sport injuries	Moderate	Moderate	High
	Most studies have adopted prospective designs but used only one measurement time-point for data collection. Stress and stress response variables have, in general, demonstrated a consistent relationship with injury risk while there has been mixed support for other categories of psychological variables (e.g. personality, coping strategies)		
Psychological risk factors for overuse sport injuries	Low/moderate	Low	High
	Few studies have investigated the relationship between psychological factors and risk for overuse injuries. There is mixed support for most of the psychological variables		
Injury risk reduction strategies	Moderate/high	High	High
	Studies have typically used RCT or quasi-experimental designs. Most studies have found reduced risk for injuries in the intervention condition		
Psychological factors influence on sport injury rehabilitation	Moderate	Moderate/high	High
	A plethora of study designs have been used; however, the majority of studies have adopted a quantitative approach using correlational and regression analyses. Frequently studied variables were pain, adherence to rehabilitation and self-efficacy which demonstrated mixed/good support		
Psychologically informed approaches to enhancing sport injury rehabilitation	Low/moderate	Low/moderate	High
	Studies have often used RCT or quasi-experimental design. Frequently used intervention programs are relaxation, guided imagery, goal setting and mindfulness. These psychological interventions have demonstrated effectiveness		
Psychological factors influencing return to sport	Low/moderate	Moderate/high	Moderate
	Most of the studies have been cross-sectional, retrospective or qualitative. Studies show consistent support for the existence of three psychological needs in returning athletes, including competence, autonomy and relatedness. An increasing number of studies demonstrate the value of meeting athletes’ psychological needs for enhancing psychological readiness to return to sport		
Psychologically informed strategies to facilitate readiness to return to sport	Low	Low	Low
	Few intervention studies have been conducted examining the efficacy and effectiveness of psychological strategies specifically aimed at facilitating psychological readiness to return. Those that have (e.g. goal setting, imagery), typically include small sample sizes and lack control groups. The efficacy and effectiveness of strategies, such as social support, reflective practice and simulation training, are typically based on qualitative or cross-sectional descriptive studies		

## Results and Recommendations

### Risk Factors for Sport Injuries

In this section, we consider the differing injury risk factors for acute and overuse injuries, highlight ways to reduce the risk of sport injury in a clinically meaningful way and summarise key recommendations for applied practice.

#### Risk Factors for Acute Injuries

Between 1998 and 2020, most research investigating psychosocial risk factors for sport injury focused on acute injuries and was framed within the revised Model of Stress and Athletic Injury [29]. According to this model an athlete’s stress response—comprised of attentional decrements (loss of sensitivity to peripheral cues and increased distractibility) and physiological changes (increased heart rate, muscular fatigue and reduced neuromuscular control)—to a potentially stressful event is hypothesised as having a direct effect on acute sport injury risk. Testing the veracity of this hypothesis, Ivarsson and colleagues [22] conducted a systematic review with meta-analysis and found that the stress response (e.g. lack of attention, concentration and decreased processing speed) has the strongest relationship with risk for acute sport injuries compared with other components of the model, namely, personality, history of stressors and coping [30–33]. One potential explanation is that strong stress responses are likely to reduce athletes' decision making capacities, leaving them vulnerable to errors, collisions and compromised motor control, thereby increasing injury risk.

Another category of risk factors proposed in the revised Model of Stress and Athletic Injury is personality traits. Much of the research on personality traits as potential injury risk factors has focused on trait anxiety and negative mood states, which have been to amplify detrimental stress responses [34–39]. While most existing research has found a positive significant relationship between several maladaptive personality factors (e.g. Type A-personality, stress susceptibility, aggression, perfectionistic concerns and athletic identity) and injury occurrence [40–43], several studies have reported inconsistent findings [44–47]. Likewise, more adaptive traits, such as optimism [48] and hardiness [49] have also been found to diminish stress responses and subsequent injury risk.

According to the model of stress and athletic injury [29], a history of stressors is a proposed category of risk factors. Research has consistently provided support for a positive relationship between injury risk and high life stress [48, 50, 51], negative life event stress [30, 31, 36, 48, 49] and daily hassles [35, 52, 53]. One potential mechanism for the link between history of stressors and injury risk is an increased magnitude of stress responses [31].

Adequate coping resources (e.g. social support) and strategies (e.g. problem focused, emotion focused) are proposed to decrease the magnitude of the stress response and are, therefore, suggested to indirectly influence the risk of injury. Cultivating strong relationships between athletes and relevant others (e.g. coaches, sport medicine providers, teammates, parents) can facilitate athlete coping and reduce stress responses [54]. Research focused on coping strategies and injury occurrence is, however, limited, and conflicting results have been reported.

Subsequent to the publication of the biopsychosocial model of stress and athletic injury and health (BMSAIH) in 2014 [55], researchers extended the scope of research on sport injury risk factors. The BMSAIH built upon the revised model of stress and athletic injury by offering understanding around mediating physiological mechanisms (e.g. stress hormone perturbation), other health conditions (e.g. illness) and behavioural influences (e.g. poor sleep) on sport injury risk. For example, both sleep quantity < 7 h/day [56] and decreased sleep volume [57] are associated with increased injury risk. In addition, psychological/lifestyle distress reported in the 7 days leading up to injury increased injury risk [56]. In support, Van der Does et al. found that in the 6 weeks leading up to injury occurrence, athletes had experienced a decrease in social recovery and general well-being [58]. Moreover, several other general well-being factors have been associated with injury risk, including symptoms of depression [59], emotional exhaustion, fatigue and decreased fitness/injury, i.e. physical stress [60]. These identified risk factors may also mediate the impact of stressors on injury risk.

#### Risk Factors for Overuse Injuries

In comparison with acute injures, fewer studies have focused on psychosocial risk factors for overuse injuries, the latter receiving greater empirical attention within the past 5 years [61]. In their recent systematic review, Tranaeus and colleagues reviewed 14 studies (9 quantitative and 5 qualitative) which reported 27 different psychosocial factors as potential overuse injury risk factors [61]. Results showed that several intrapersonal factors, such as personality traits (e.g. perfectionistic concerns, obsessive passion), previous injuries and neglecting bodily warning signals, had a potential effect on the risk of overuse injury. Several interpersonal and sociocultural factors, such as poor coach-athlete relationships, lack of social support and pain normalisation, were also identified as potential injury risk factors. All these identified psychosocial sport injury risk factors can, often in combination with an extensive training load, increase the chances of athletes engaging in maladaptive behaviours (e.g. overtraining). If an athlete repeatedly engages in these types of behaviours the likelihood of sustaining an overuse injury is increased.

#### Injury Risk Reduction Strategies

Research into the role of psychological interventions in clinically meaningful injury risk reduction has been established over the past 30 years [22, 62]. Given the relationships between psychological stress and acute injury risk, much of the research in this area has centred on interventions that help individuals manage the impact of this stress. Interventions with a stress management focus (e.g. mindfulness and acceptance-based practices) have consistently been shown to result in a clinically meaningful injury risk reduction (Table 2).

**Table 2:** Psychological interventions to reduce sport injury risk, with the most recent articles listed first

Study	Participants	*N* (M/F, age range or mean age, years)	Intervention	Results
Naderi et al. (2020) [67]	Elite male soccer players	168 (168/0, 16–19)	Mindfulness (MAC approach)	Number of injuries, average injuries per team and days lost owing to injury were lower in the mindfulness group than the control group
Zadeh et al. (2019) [68]	Amateur male soccer players	45 (45/0, 24.3)	Mindfulness (MAC approach)	Fewer injuries in the intervention group (0.45 injuries/player) than the control group (2.82 injuries/player)
Olmedilla-Zafra et al. (2017) [69]	Male soccer players	74 (74/0, 17–19)	Stress inoculation therapy (PMR, breathing, imagery, self-instructional and attention-focus training)	Fewer injuries in the intervention group (0.11 injuries/player) than the control group (0.24 injuries/player)
Tranaeus et al. (2015) [70]	Male and female elite floorball players	346 (174/172, 23.4)	Stress management, relaxation, goal setting skills and emotional control	Fewer injuries in the intervention group (0.49 injuries/player) than the control group (0.53 injuries/player)
Tranaeus et al. (2015) [71]	Male and female elite floorball players	401 (203/198, 22.4)	Stress management, relaxation, goal setting skills and emotional control	Fewer injuries in the intervention group (0.31 injuries/player) than the control group (0.41 injuries/player)
Ivarsson et al. (2015) [72]	Male and female junior elite soccer players	41 (41/0, 16–19)	Mindfulness (MAC approach)	Fewer injuries in the intervention group (8 injuries) versus the control group (15 injuries)
Edvarsson et al. (2012) [73]	Male and female high school soccer players	29 (22/7, 16–19)	Self-regulation techniques	Fewer injuries in the intervention group (0.38 injuries/player) than in the control group (1.0 injuries/player)
Noh et al. (2007) [74]	Female ballet dancers	35 (0/35, 14–19)	Autogenic training, broad-based coping skills (AT, imagery and self-talk)	Overall reduction in injury burden in the intervention group Broad-based coping skills most effective at reducing injury risk
Maddison and Prapavessis (2005) [75]	Rugby players	48 (48/0, 16–34)	CBT	Fewer injuries in the intervention group (2.84 injuries/athlete) than the control group (3.43 injuries/athlete)
Kolt et al. (2004) [76]	Male and female gymnasts	20 (3/17, 10–25)	Cognitive-behavioural stress management	Fewer injuries in the intervention group (2.5 injuries/athlete) than the control group (3.2 injuries/athlete)
Kerr and Goss (1996) [77]	Gymnasts	24 (16/8, 14–25)	Stress inoculation training	Fewer injuries in the intervention group than the control group (Cohen’s *d* = 0.67)
Davis (1991) [78]	Swimmers and football players	21 (NA)	Relaxation training and imagery	A 52% decrease in injuries for swimmers and 33% decrease in injuries for football players

*M* male, *F* female, *NA* not announced, *MAC* mindfulness-acceptance-commitment, *PMR* progressive muscle relaxation, *AT* autogenic training, *CBT* cognitive behavioural training

Both the efficacy and effectiveness of the different interventions have been demonstrated across noted systematic reviews [22, 62]. Importantly, all experimental studies to date demonstrate a smaller number of injuries in intervention groups versus control groups (Table 2). Hence, intervention use with athletes should be considered a central element of athlete care if seeking to reduce the risk of sport injuries.

Whilst most of the existing evidence-base has centred on stress management interventions that athletes can use themselves (e.g. mindfulness and acceptance), there has also been recent psychological research that focuses on cultural narratives [63] and associated resources [64] that could further help to lower injury risk. For example, one of the dominant cultural narratives in sport is the culture of risk that downplays health and safety and encourages athletes to play through and with pain and injury [65, 66]. However, Everard et al. [63] recently identified a counter cultural narrative (i.e. longevity narrative) that encourages athletes to ‘train smart, not hard’ and to develop a more compassionate body-self relationship, which ultimately works towards and promotes more sustainable careers in elite sport.

#### Recommendations for Applied Practice in Injury Risk Reduction

Combining research on injury risk factors and injury risk-reduction strategies, we offer four key applied recommendations for those seeking to reduce athletes’ risk of sport injuries:

- Form strong relationships with your athletes and colleagues. Common factors, such as shared goal consensus/collaboration, empathy, working alliance and positive regard are all important for open dialogue regarding injury and injury risk factors.
- Screen athletes frequently and consistently for psychosocial stress indices, sleep quality, and perceived recovery (e.g. using the Hooper Index). Use this screening to inform open communication regarding athletes’ experiences of injury risk factors.
- Consider education around psychosocial injury risk factors for coaches, sports medical staff and athletes. This education could include organisational culture, psychological safety, psychosocial stressors and relational issues. Such education could contribute to reducing athlete stressors and mitigating against poor behavioural choices, thus reducing overuse injury risk.
- Adopt mindfulness and acceptance-based practice and stress management approaches to reduce acute injury risk.

### Psychological Factors Influencing Sport Injury Rehabilitation

In this section, we describe research on individual and interpersonal psychological factors influencing sport injury rehabilitation and highlight psychologically informed approaches to enhance rehabilitation experiences. Broad psychological themes evident post-injury include stressors, coping, psychological adaptation and adjustment and psychological interventions in rehabilitation.

#### Individual Factors Influencing Sport Injury Rehabilitation

A substantial amount of research investigating psychological factors influencing sport injury rehabilitation has focused on acute injuries and has been framed within the integrated model of psychological response to the sport injury and rehabilitation process [79]. The vast majority concerns individual or intrapsychic factors (i.e. processes within the mind [80]) influencing rehabilitation and fall into three categories: cognitions (e.g. motivation), emotions (e.g. fear of reinjury) and behaviour (e.g. rehabilitation adherence).

Early research on cognitive appraisals and cognitions in response to sport injury focused on themes such as athletic identity, perceptions of loss, attributions for injury causality and pain catastrophizing [15]. Research continues to explore similar broad themes related to athlete personality, motivation and confidence and their influences on sport injury rehabilitation. Psychological factors, such as hardiness [49], resilience [81] and flourishing [82], all demonstrated relationships with desirable post-injury psychological responses; however, several of these factors reveal wide individual differences [83]. Research also shows that high self-efficacy to facilitate recovery is a useful cognitive factor to facilitate recovery among both senior [84] and junior [85] athletes. Studies show that a less favourable individual factor to cope with the rehabilitation period is severe pain catastrophizing and its association with depressive symptoms [86] and negative association with return to a similar level of sport [87]. Patient motivation is related to a favourable rehabilitation environment, patient satisfaction and returning to the pre-injury sport activity [88, 89]. A strong athletic identity has been associated with increased risk of post-injury psychological distress [86], not least to those for whom sport is central to lifestyle and personal identity, such as young athletes [90]. Finally, studies show that individual factors, such as perfectionism, influence the coping techniques [91] and mental health [92] of injured athletes and affect the course of rehabilitation differently depending on the type of perfectionism that is activated.

Formative research on emotional or affective responses to injury prior to 2011 primarily has shown how psychological factors, such as mood disturbances (e.g. fatigue, frustration), mental health concerns (e.g. depression and anxiety) and fears (e.g. about reinjury, pain and movement) were detrimental to athletes’ rehabilitation experiences [15]. Recent research has also highlighted the benefits of positive psychological factors, such as optimism [48], gratitude [93], self-compassion [94], sport injury related growth [95] and spirituality [96], in adaptive coping and well-being during sport injury rehabilitation. Injury and performance-related fears are associated with rehabilitation outcomes [23] and can lead to poor rehabilitation outcomes [97], and from a long-term perspective, lower levels of fear appear to be favourable [98].

Behavioural factors influencing rehabilitation and recovery explored in research prior to 2011 include rehabilitation under- or over-adherence, exercise dependence, help seeking and steroid or nutritional supplement use [15]. Rehabilitation adherence and help-seeking continue to be important behavioural topics along with fear avoidance. Psychological factors including motivation, confidence/self-efficacy, coping, social support and locus of control [99, 100] influence behaviours, such as adherence to rehabilitation, and rehabilitation adherence has a positive effect on outcomes, such as return to sport [22]. Findings also demonstrate that negative affectivity, high athletic identity, and self-presentational concerns are associated with rehabilitation over-adherence (i.e. engaging in excessive rehabilitation [100]). Those with higher fear avoidance [101] and lower perceived athletic ability post-injury [102] may experience greater pain. A negative attitude to help-seeking and accessibility issues have been identified as key barriers to mental health help-seeking among elite athletes with a history of moderate or severe injury [102].

#### Interpersonal Factors Influencing Sport Injury Rehabilitation

Alongside the individual factors influencing rehabilitation, interpersonal or interpsychic factors [80] play a central role in understanding communication and interaction patterns between the injured athlete and their immediate social environment (e.g. physiotherapist, doctor, coach and family members). Social support seeking, use of social networks and social influences from coaches and others are all important for enhancing sports injury rehabilitation [15, 103].

A considerable body of research indicates that injured players with high perceptions of social support from significant others (e.g. athletic trainers) will experience greater satisfaction and less anxiety and depression during the rehabilitation period [59, 104–106]. A lack of social support from the team and the coach negatively interferes with the rehabilitation [107] which underscores the importance of comprehensively examining the multiple constructs and providers of social support [108].

Being a key-player in the rehabilitation of injured athletes, it is reported that physiotherapists use a rather small number of behaviour-change techniques [109] but also show an appreciation for incorporating additional psychological interventions within their practice [16]. To help improve current best practice, physiotherapists encourage researchers to develop psychologically centred interventions for rehabilitation [110].

Injured athletes’ psychological experiences, including their thoughts, feelings and behaviours, are affected by broader sociocultural factors, such as institutional and global forces [15, 111]. Examples of these influences include social norms for playing through pain and injury and the ethos of sacrificing health in pursuit of high performance [15].

#### Psychologically Informed Approaches to Enhancing Sport Injury Rehabilitation

Psychologically based strategies to better understand and help injured athletes during rehabilitation are central to creating the best conditions for a multidisciplinary rehabilitation plan. Literature prior to 2011 included a small number of studies reporting generally positive benefits resulting from psychological interventions in rehabilitation, such as imagery, relaxation, goal setting and solution focused brief counselling [15]. A 2012 review of the effectiveness of psychological intervention following sport injury [112] found robust support for associations between guided imagery/relaxation, improved psychological coping and reduced re-injury anxiety. More recently, versions of mindfulness and acceptance-based practice as well as cognitive-behavioural based intervention programs have predominated. Table 3 summarises research on psychologically informed intervention approaches to enhance rehabilitation experiences among athletes.

**Table 3:** Psychological intervention studies to enhance rehabilitation experiences among injured athletes (*n* = 428), with the most recent articles listed first

Study	Participants	*N* (M/F, age range or mean age, years)	Intervention	Results
Brewer et al. (2022) [113]	Competitive and recreational athletes experiencing ACL surgery	69 (39/30, 15–67)	Interactive cognitive-behavioural multimedia program	Intervention group showed greater preoperative confidence in ability to cope, lower postoperative pain and kinesiophobia, and greater use and perceived utility of patient education materials
Bagheri et al. (2021) [114]	Female runners with patellofemoral pain	30 (0/30, 28.3)	Mindfulness program	Intervention group showed decreased pain intensity, fear of reinjury, and pain catastrophizing, and improved knee function and pain coping
Salim and Wadey (2021) [93]	Athletes in return to sport phase of sport related injury	30 (19/11, 18–32)	Gratitude intervention	Intervention group benefited most in the “relating to others” dimension of sport-injury related growth
Coronado et al. (2020) [115]	Athletes experiencing ACL surgery	8 (2/6, 15–22)	Cognitive-behavioural based physical therapy intervention	Intervention delivered via telephone appears to be a feasible approach for addressing psychological risk factors in rehabilitation
Moesch et al. (2020) [116]	Elite or sub-elite athletes with serious injuries	6 (2/4, 18–39)	Mindfulness- and acceptance-based intervention	Intervention group showed improvements in nonreactivity and well-being
Podlog et al. (2020) [117]	Collegiate Division I athletes sustaining injury requiring a minimum 4-week absence from training or competitive performance	16 (4/12, 19.9)	Cognitive-behavioural intervention	Intervention group reported more positive emotions and reduced negative emotions over the course of rehabilitation
Rollo et al. (2017) [118]	Athletes who sustained a moderate to severe musculoskeletal sport injury and underwent rehabilitation	28 (19/9, 18–36)	Heart rate variability biofeedback training	Intervention showed promise in improving pain catastrophizing and the psychological responses of injured athletes throughout the rehabilitation process
Salim and Wadey (2018) [119]	Competitive athletes from individual and team sports who had returned to sport following serious injuries	45 (28/17, 18–40)	Emotional disclosure	Intervention (verbal disclosure) group experienced sport injury related growth
Shapiro and Etzel (2018) [120]	Collegiate soccer, wrestling and cheerleading athletes	5 (4/1, 19.0)	Four mental skills (goal setting, relaxation, imagery, managing self-talk)	Interventions led to several positive physical and psychological consequences
Coppack et al. (2012) [121]	Military personnel in rehabilitation for lower back pain	48 (45/3, 18–48)	Goal setting	Intervention group showed higher rehabilitation adherence and self-efficacy
Mahoney and Hanrahan (2011) [122]	Competitive athletes from soccer, squash and sailing	4 (2/2, 18–49)	Acceptance and commitment therapy	The intervention helped injured athletes commit to rehabilitation protocols and behaviours
Mankad and Gordon (2010) [123]	Injured athletes, elite level	9 (4/5, 18–29)	Written emotional-disclosure intervention	Athletes feeling less devastated, dispirited, cheated, and restless by their injury and increasing the reorganization of their thoughts
Evans and Hardy (2002) [124]	Injured athletes separated across three groups	39 (33/6, 17–39)	Goal setting intervention	Greater rehabilitation adherence and self-efficacy, and lower levels of dispiritedness
Rock and Jones 2002 [125]	Injured athletes, conservatively treated ACL injuries	3 (2/1, 31–40)	Counselling intervention	Outcome measures; mood, perceived rehabilitation, pain ratings, and social support, provided some evidence of the beneficial impact of counselling skills on psychological outcomes
Cupal and Brewer (2001) [126]	Competitive and recreational athletes	30 (16/14, 18–50)	Relaxation, guided imagery	The intervention led to lower levels of reinjury anxiety and pain compared to the placebo and control groups
Johnson (2000) [127]	Injured athletes, competitive level	58 (52/6, 23.7)	Goal setting, stress management, and imagery	Improved mood and perceived readiness to return to competition
Brewer et al. (1994) [128]	Injured athletes who underwent rehabilitation for ACL injuries	20 (12/8, 25.1)	Goal setting, imagery, and counselling	Psychological interventions were perceived positively in the context of sport injury rehabilitation by injured athletes

*M* males, *F* females, *ACL* anterior cruciate ligament

#### Recommendations for Applied Practice in the Psychology of Sport Injury Rehabilitation

Drawing on our review of literature, we offer three recommendations for applied practice among those seeking to use psychology to enhance the rehabilitation experiences of injured athletes (e.g. coaches, practitioners within multidisciplinary teams and athletes themselves):

- Incorporate elements of mindfulness and acceptance-based practices and cognitive-behavioural-based programs to improve coping and well-being. These elements focus on improving awareness and acceptance of one’s current thoughts and feelings as a basis for psychological flexibility and positive action towards recovery.
- Provide time and space to contribute as much social support as possible. Athletes’ sense of support stems from feeling heard and cared for, and this is important throughout the rehabilitation process.
- Cultivate elements of a positive psychology mindset, such as optimism, gratitude, and self-compassion, to benefit mood and promote sport injury-related growth. These elements aid athletes in focusing on seeing meaning and possibilities beyond their present circumstances and beyond themselves.

### Psychological Factors Involved in Return to Sport

Research on psychological factors involved in the return to sport (RTS) phase has primarily been undertaken within the past 20 years. Much of the impetus for such research lies with the recognition that physical and psychological readiness to RTS do not necessarily coincide [129]. Calls for more holistic assessment of the myriad factors influencing athletes’ ability to successfully RTS following injury have also led to greater efforts to assess this transition period. Importantly, the transition from rehabilitation to the sport environment can involve multiple steps and pathways via which some athletes return to competitive activities while others do not. This section of the consensus statement synthesises the evidence to outline: (a) various RTS trajectories; (b) athletes' psychological needs for autonomy, competence and relatedness; and (c) psychologically informed strategies that can enhance readiness to RTS.

#### What are Various Return to Sport Trajectories?

For many athletes—and practitioners working with them—their aim is to return to their pre-injury level of functioning. Aligned with this trajectory, Taylor and Taylor [129] proposed a five-stage physical and psychological RTS model (i.e. initial return, recovery confirmation, return of physical and technical abilities, high-intensity training and return to competition). More recently, Ardern et al. [14] proposed a three-stage process involving a return to participation, a return to sport and a return to competition. Shifting away from models, Mainwaring [130] constructed a theory on the basis of a prospective longitudinal examination of athletes who had sustained an ACL rupture. Her findings revealed how athletes were largely concerned with restoring their sense of self and overcoming injury to return to their pre-injury level of functioning. Yet, while this trajectory is the most commonly embedded within elite sporting cultures [63] and the most heavily researched [131], athletes can experience other trajectories: (a) returning to sport beyond their pre-injury level of functioning (e.g. increased resilience, improved sporting performance), (b) returning to sport below their preinjury level of functioning (e.g. reduced self-confidence, lowered performance level), (c) returning to sport and becoming re-injured or experiencing a related sporting injury, and (d) retiring from formal sport because of being unable to return (e.g. career ending injury) or choosing not to return, which might or might not involve a transition to physical activity [132–135]. Which trajectory athletes work towards and ultimately attain depends on a multiplicity of factors: the nature of their physical injury, the physical environment and socio-cultural climate in which they attempt their RTS and the extent to which they experience satisfaction of their psychological needs [136].

#### Athletes’ Psychological Needs During Return to Sport

When navigating various RTS trajectories, athletes commonly experience three psychological needs: competence, autonomy, and relatedness [132, 136]. Competence pertains to athletes’ sense of proficiency in their sporting capabilities and is epitomised by apprehensions regarding one’s ability to achieve their fullest athletic potential, to achieve and/or surpass pre-injury performance levels, to navigate anxiety about re-injury and self-presentational concerns about meeting the performance expectations of relevant others [132, 136–138]. Autonomy pertains to the extent to which injured athletes experience a sense of control over their desired RTS trajectory [139]. Athletes who are internally motivated to return for reasons of their own choosing (e.g. love of the sport, a desire to interact with teammates and contribute to team success) are considered autonomous (i.e. volitional) in their RTS. However, the evidence illustrates that many athletes experience a lack of autonomy in their return to sport [140]. For example, external and internal pressures to RTS within a particular time frame or RTS for a variety of non-autonomous reasons, such as worries about being replaced on the team, the need to maintain financial benefits (e.g. scholarships, salaries) associated with sport involvement or pressures from coaches or teammates to help one’s team achieve victorious outcomes [132, 137]. Finally, the notion of relatedness pertains to feelings of social connection and affiliation. Relatedness issues have been identified as taking the form of a lack of appropriate social support or the belief that one is not a “full” team member given athletes' restricted participation while attempting to reintegrate into their sport [132, 137].

The extent to which athletes experience fulfilment of these three psychological needs will influence their psychological readiness and ultimately their RTS trajectory [138, 140]. For instance, Wadey et al. [131] found that athletes who coped with their re-injury anxieties reported successful RTS outcomes. Conversely, athletes who did not address competence, autonomy and relatedness issues were found to return at lower competitive levels, withdraw from sport or experience a re-injury [132, 141]. For example, meta-analytic data suggest that re-injury concerns were the most frequently cited reason for reduction in sports participation, with only 63% of athletes returning to pre-injury levels, despite 85% achieving clinically satisfactory outcomes [142]. Re-injury concerns typically involve reductions in perceived competence (e.g. ‘I’m no longer sure my body can handle the demands of competitive sport or if I get re-injured it will be even more difficult to attain desired levels of athletic proficiency’), autonomy (e.g. ‘I no longer feel in control of my body’s ability to stay healthy or to avoid re-injury’) and relatedness (e.g., ‘If I get re-injured I’ll once again be removed from the sport environment and people I care about and/or who reinforce my identity as an athlete’). Re-injury concerns delay or prevent a return to sport, increase attentional distraction, and negatively affect athletes’ post-injury performances [142, 143].

#### Psychosocially Grounded Strategies to Facilitate Readiness to Return to Sport

A critical question of importance for athletes and sport injury stakeholders is how to foster physical and psychological readiness to RTS [144, 145]. Multiple evidence-based strategies have been identified that can bolster athletes’ competence, autonomy and relatedness [137, 140] and thereby facilitate psychological readiness. It is recommended that athletes focus on building confidence that their injury has recovered and can withstand the demands of competitive sport. Evidence-based strategies include goal setting, self-talk, imagery, emotional support, informational support, reflective practice and simulation training [103, 132, 137, 146]. Examples of these strategies include coaches simulating matches with lower calibre opponents for athletes returning to sport to build their confidence [137], and the use of physical practice (e.g. static tackling with tackle bags to simulate training), mental imagery (e.g. images of the athlete withstanding threatening situations to enhance self-confidence) and the use of process goals to help focus athletes’ attention on task-related activities [146].

Collaboration between athletes and their coaches and medical team is essential to ensure an effective RTS [144]. For example, informational support can also help athletes to avoid returning prematurely and increasing their risk of re-injury [132, 137]. This support should be received from medical staff, coaches and others with injury specific knowledge and experience. Further, its content should focus on encouraging athletes not to rush their return and explicit instructions regarding what the athletes can and cannot do to facilitate their return. Sport psychologists can facilitate the provision of this informational support by enabling clear lines of communication between athletes, coaches and medical staff that meet athletes’ needs [137, 140]. However, for those athletes who experience a career-ending injury and are unable to return to sport, it might be that they mobilise other members of their sport network (e.g. performance lifestyle advisors) to support their mental health and promote a successful transition to another role in sport (e.g., coaching) and/or away from sport [147].

#### Recommendations for Applied Practice in the Psychology of Return to Sport

- Utilise strategies that facilitate athletes’ sense of competence (goal setting, positive self-talk, imagery and reflective practice), autonomy (athlete input into RTS dates, reduce pressures to return and provide choice in level of team/sport involvement) and relatedness (informational and emotional support, inclusion in appropriate team activities and one-on-one meetings with significant others).
- Help injured athletes to progressively simulate the competitive environment.
- Facilitate clear lines of communication between athletes, coaches, and medical staff to ensure that athletes are psychologically ready.

## Discussion

### Future Directions: Where Will the Next 50 Years Take Us?

In summarising 50 years of psychology of sports injury research, it is evident that a robust evidence-base exists regarding the role of psychological factors in injury onset, rehabilitation and return to sport. In examining this body of work it is evident that the psychology of sport injury research has remained largely siloed, and that future quantitative, qualitative and mixed methods research needs to be more rigorous (e.g. injury reporting). While increasing evidence of interdisciplinary work exists [148], we believe that greater collaboration between various scientific and clinical disciplines (e.g. sports medicine, sociology, psychology and coaching science) is needed to enhance understanding of pertinent issues and to better address real-world challenges. We also advocate moving beyond traditional biopsychosocial approaches to sport injury, to include a nuanced understanding of institutional (physical environment, psychosocial architecture), socio-cultural (e.g. norms, collective values, cultural narratives) and policy (e.g. national, governing sport-body policies) level factors. Towards this end, adopting multilevel sport injury models [8] has the potential to provide a more comprehensive and nuanced picture of athlete injury experiences as well as interactions between athletes and a range of sport injury stakeholders. For instance, institutional level factors, such as run-down facilities or poor field conditions, may increase injury vulnerability. Once injured, sociocultural factors (e.g. cultural narratives about demonstrating resilience by making a quick recovery) may influence athletes to try to expedite their rehabilitation. Finally, athletes may interact with various injury stakeholders (coaches, physicians, physiotherapists) as they navigate policies and procedures regarding safe RTS.

Despite the evident challenges in integrating sport psychology practice into mainstream athlete care, doing so may facilitate enhanced athlete care and mitigate potential liability issues. Stakeholder conversations with an open, trusting, interdisciplinary focus would be central to this development.

### Strengths and Limitations

Although this consensus statement is not a systematic review, the statement nonetheless includes pertinent evidence needed to provide clinical recommendations. Several limitations, however, are noted. First, the consensus statement only covers scientific literature published in English. Second, while efforts were made to foster a diverse and inclusive group of experts, our authorship team did not include individuals from the Southern Hemisphere. Along these lines, much of the research highlighted was based in the Northern Hemisphere and focussed on able-bodied athletes. Certainly, scholarship from the global south, including South American, Asian and African nations, and particularly countries with lower gross domestic products, is warranted. Final limitations were that this statement did not include guidelines for concussion, and nor did we provide recommendations for athletes in parasports. In instances where recommendations in this consensus statement are not applicable, specific guidelines may be needed for various rehabilitation contexts or injured populations.

## Conclusions

All sports medicine practitioners are committed to improving the health and well-being of athletes. Sport injury represents an existential threat to such health and well-being. As demonstrated, psychological considerations are important elements of an athlete’s injury, whether prior to or following injury occurrence. Hence, psychological factors should be considered on par with and in conjunction to physical, physiological or biomechanical injury considerations. In critically evaluating 50 years of psychology of sport injury research, this group has advanced an understanding of psychological considerations in applied injury management across the injury lifespan. Hopefully this consensus statement can inform guidelines for gold standard, interdisciplinary injury care over the next 50 years and beyond.

## Footnotes

1 This review does not account for the psychology of sport concussion. For readers interested in this field, we encourage them to read the book Bloom and Caron [17] and position paper Bloom et al. [18].
